# Heart rate response to transient hypoxia in patients with heart failure and Cheyne–Stokes respiration

**DOI:** 10.1113/EP092304

**Published:** 2025-02-17

**Authors:** Gian Domenico Pinna, Elena Robbi, Maria Teresa La Rovere, Roberto Maestri

**Affiliations:** ^1^ Department of Biomedical Engineering of Montescano Institute Istituti Clinici Scientifici Maugeri IRCCS Montescano Italy; ^2^ Respiratory Physiopathology and Sleep Unit of Montescano Institute Istituti Clinici Scientifici Maugeri IRCCS Montescano Italy; ^3^ Department of Cardiology of Montescano Institute Istituti Clinici Scientifici Maugeri IRCCS Montescano Italy

**Keywords:** cardiac reflex, hypoxic ventilatory response, oxygen desaturation, pulmonary inflation reflex, tachycardia

## Abstract

Cheyne–Stokes respiration (CSR), a rhythmic rise and fall in ventilation often experienced by patients with heart failure during sleep, is typically accompanied by an oscillation in heart rate (HR) at the same frequency. The mechanisms responsible for this oscillation are still debated. In this study, we used the experimental model of the transient hypoxia test (i.e., a laboratory test that mimics the transient nature of the cyclic desaturations that occur during hyperpnoeic phases of CSR) to assess accurately the temporal relationship between the HR response to transient hypoxia and the tidal volume response in six heart failure patients. The same relationship was assessed during CSR using polysomnographic signals. We hypothesized that this relationship would provide important insights into the key mechanisms contributing to the HR response. During transient hypoxia, HR started to increase around the onset of tidal volume increase but continued to increase after the peak of the latter had been reached. The time delay between the two peaks (HR vs. tidal volume) was 7.9 ± 4.8 s. The same delay during hyperpnoeic phases of CSR was 1.0 ± 0.9 s. In addition, the increases in lung volume were much greater than those found in the laboratory tests. Based on the known dynamics of vagal and sympathetic control of HR, we speculate that the HR response to transient hypoxia might be attributable predominantly to the sympathetically mediated tachycardic effect of the increased central inspiratory drive, whereas the fast, vagally mediated pulmonary inflation reflex might be the predominant mechanism during CSR.

## Abstract (highlights)

- **What is the central question of this study?**
  Are the key mechanisms underlying the cyclic increase in heart rate during intermittent transient hypoxia of Cheyne–Stokes respiration and during experimental transient hypoxia the same?
- **What is the main finding and its importance?**
  During experimental transient hypoxia, heart rate peaked significantly later relative to the peak in lung volume, whereas during Cheyne–Stokes respiration the peaks in heart rate and lung volume were almost synchronous. This study suggests that the key mechanisms underlying the increase in heart rate during the transient hypoxia of Cheyne–Stokes respiration and during experimental transient hypoxia are different.

## 1 INTRODUCTION

According to the largest study to date (Oldenburg et al., 2016), a significant proportion (∼40%) of patients with heart failure and reduced left ventricular systolic function experience rhythmic waxing and waning of ventilation during sleep, characterized by periods of hyperpnoea alternating with central apnoea or hypopnoea, commonly referred to as Cheyne–Stokes respiration with central sleep apnoea (CSR‐CSA) (Yumino & Bradley, 2008). This oscillatory breathing pattern is typically accompanied by an oscillation in heart rate (HR) at the same frequency (Leung et al., 2003; Pinna et al., 2021).

Tachycardia is a well‐known effect of acute hypoxia (Daly, 1986; Marshall, 1994). Given that the rhythmic increase in ventilation during CSR‐CSA is caused primarily by the stimulation of carotid chemoreceptors by oscillating arterial partial pressures of CO_2_ and O_2_ ($P_{a}CO_{2}$ and $P_{a}O_{2}$) (Cherniak & Longobardo, 1986; Khoo et al., 1991, 1982), transient dips in oxygen saturation at these chemoreceptors have been proposed as a mechanism contributing to the cyclic increase in HR. However, based on their observation that the oscillation in HR persisted even when intermittent hypoxia was eliminated by overnight O_2_ inhalation, Leung et al. (2003) speculated that it is the cyclic increase in ventilation, rather than the concomitant dips in oxygen saturation, that induces the cyclic increase in HR during CSR‐CSA. We felt that this hypothesis should be treated with caution because, on the one hand, $P_{a}CO_{2}$ was still oscillating at the carotid chemoreceptors during O_2_ inhalation and was, therefore, a potential confounder, and on the other hand, the mechanisms mediating the HR response to transient oxygen desaturations alone had not been investigated.

In this study, we investigated these mechanisms using the experimental model of the transient hypoxia test, one of the methods used to assess the hypoxic ventilatory response in humans (Edelman et al., 1973). This test mimics the transient nature of the cyclic desaturations that occur overnight during CSR‐CSA, and it is easy to perform and well tolerated by patients. In addition, because it is performed in the laboratory, it allows accurate monitoring and analysis of the time course of HR relative to that of lung volume and ear oxygen saturation, the latter being a delayed, reasonably accurate estimate of oxygen saturation at the carotid chemoreceptors. Subprimate studies have consistently shown that the cardiac reflex elicited by the activation of pulmonary stretch receptors by increased ventilation produced by carotid chemoreceptor stimulation is a major mechanism contributing to the tachycardic response to hypoxia and is predominantly vagally mediated (Daly, 1986; Marshall, 1994). Therefore, given that the HR response to a change in cardiac efferent vagal activity is very fast (Katona et al., 1970), the occurrence during the transient hypoxia test of a significantly delayed HR increase relative to the associated increase in lung volume (i.e., an index of lung stretch) would rule out a dominant contribution from lung stretch receptors. This hypothesis was tested in the first part of the study. In the second part, we performed a similar analysis of ventilatory parameters and HR on polysomnographic signals recorded overnight during CSR‐CSA.

## 2 MATERIALS AND METHODS

### 2.1 Ethical approval

The Central Ethics Committee of the Salvatore Maugeri Foundation granted ethical approval for this study (CEC, # 716). All patients gave written informed consent to participate in the study. All procedures were performed in accordance with the tenets of the *Declaration of Helsinki* (World Medical Association, 2013).

### 2.2 Subjects

The study was conducted between 2012 and 2016 in consecutive hospitalized patients with chronic heart failure (HF) who were part of an investigation into the individual pathophysiological mechanisms of CSR‐CSA. Assessment of the hypoxic ventilatory response was part of the investigation. Inclusion criteria were as follows: (1) stable clinical condition and optimal medical treatment; (2) left ventricular ejection fraction of <45%; (3) New York Heart Association functional class II–III; and (4) dominant CSA with apnoea–hypopnoea index (AHI) of ≥15/h. Patients were excluded if they had chronic obstructive pulmonary disease or cognitive impairment.

### 2.3 Assessment of the hypoxic ventilatory response

The hypoxic ventilatory response was assessed using the transient hypoxia method originally proposed by Edelman et al. (1973) and subsequently adapted and widely used by several investigators in HF patients and healthy subjects (Chua et al., 1997; Niewinsky et al., 2013; Paleczny et al., 2019; Tubek et al., 2016). All tests were performed between 10:00 and 12:00 h in a quiet laboratory and a seated position, using a custom‐made system (Maestri et al., 2013). A detailed description of the test can be found in the Appendix.

### 2.4 Assessment of the HR response to transient hypoxia

The HR response to transient hypoxia was assessed in the two most desaturating trials of those with an oxygen saturation nadir of <90% (i.e. at least moderate hypoxaemia), provided that sinus rhythm was present throughout the duration of these trials. Given that this assessment involves measuring the temporal relationship between $V_{T}$ and beat‐to‐beat HR and between $\dot{V}$_I_ and $S_{p}O_{2}$, all of which except $S_{p}O_{2}$ are recorded at unequally spaced time points, we calculated a 10 Hz resampled version of the $V_{T}$, $\dot{V}$_I_ and HR time series using a cubic smoothing spline, where this is the sampling frequency of $S_{p}O_{2}$. The result was four signals sampled at the same frequency. In addition, the oxygen saturation signal at the carotid chemoreceptors was estimated from the $S_{p}O_{2}$ signal recorded at the ear. A detailed description of the procedure can be found in the Appendix.

For each of the two tests, the change in HR, $\dot{V}$_I_ and $V_{T}$ induced by the hypoxic stimulus was calculated as the difference between the peak of the corresponding signal and its mean baseline value. Likewise, the change in estimated $S_{p}O_{2}$ was calculated as the difference between its nadir and its mean baseline value. The delay between the peak of HR and that of the respiratory signals was also calculated. Finally, the mean of the results of the two tests was calculated.

### 2.5 Assessment of relevant ventilatory, oxygen saturation and HR parameters during CSR‐CSA

Unattended standard polysomnography was performed between 21:30 and 07:00 h in the patient's own bed using a portable polysomnograph (Embla Titanium, Embla Systems, Thornton, CO, USA). Sleep studies were scored visually according to the recommendations of the American Academy of Sleep Medicine. The scoring information and raw polysomnographic signals were then transferred to a dedicated workstation for subsequent analysis using software developed in house.

An uncalibrated lung volume signal was obtained by summing the thorax and abdomen movement signals obtained by respiratory inductance plethysmography. This signal was used to calculate an estimate of the mean change in lung volume during CSR‐CSA (= mean peak lung volume during the hyperpnoeic phases minus mean baseline lung volume during normal breathing) for each patient. In addition, the mean $S_{p}O_{2}$ nadir associated with the lung volume peaks, and the mean delay between the lung volume peaks and the corresponding HR peaks were calculated. Details can be found in the Appendix.

### 2.6 Statistical analysis

Results are summarized as the mean ± SD and/or median (quartile 1, quartile 3) where appropriate. The relationship between variables was assessed using Spearman's rank correlation. A value of *P* < 0.05 was considered statistically significant.

## 3 RESULTS

Of the 20 patients who underwent assessment of the hypoxic ventilatory response, one was excluded from the analysis because his baseline $S_{p}O_{2}$ was 90% and another because he had back pain during the test. Eleven patients were not in sinus rhythm during the test (two had atrial fibrillation, and the rest had a permanent pacemaker or implantable cardioverter defibrillator (ICD; the patients with ICDs had occasional paced beats during some of the tests, despite being in sinus rhythm at baseline). One patient had a large number of ventricular ectopic beats. The mean age, body mass index and left ventricular ejection fraction of the six patients included in the study were 59.6 ± 8.2 years, 28.3 ± 2.7 kg/m^2^ and 30.2% ± 6.3%, respectively. Three patients were in New York Heart Association class II, and the remaining were in class III. All patients were taking: (1) an angiotensin‐converting enzyme inhibitor or an angiotensin receptor blocker; and (2) a β‐blocker.

### 3.1 Hypoxic ventilatory response

Individual values and summary statistics of the hypoxic ventilatory response are shown in Table 1 (alternative summary statistics are presented in Table A1 in the Appendix). The $S_{p}O_{2}$ nadir observed during the test with the greatest desaturation was almost equal to the minimum $S_{p}O_{2}$ observed during sleep in the same population of patients (Pinna et al., 2024). The mean hypoxic ventilatory response was higher than that previously reported in normal subjects (Chua et al., 1997; Paleczny et al., 2019), lower than that reported in early studies in HF patients with left ventricular dysfunction (Chua et al., 1997), but similar to that observed more recently in these patients (Niewinsky et al., 2013).

**TABLE 1:** Individual hypoxic ventilatory response data.

Patient no.	HVR (L/min/%$S_{p}O_{2}$)	$S_{p}O_{2}$ nadir (%)	$\dot{V}$_I_ peak (L/min)
1	−0.62	87.2	16.5
2	−0.33	82.9	20.4
3	−0.72	85.9	15.9
4	−0.47	88.0	13.5
5	−1.03	84.9	18.4
6	−0.22	83.7	14.6
Mean ± SD	−0.57 ± 0.29	85.4 ± 2.0	16.6 ± 2.5

*Note*: The individual oxygen saturation nadir and corresponding peak ventilation during the nitrogen breathing test with the greatest desaturation are also given.

Abbreviations: HVR, hypoxic ventilatory response; $S_{p}O_{2}$, oxygen saturation by pulse oximetry with an ear probe $;\;\dot{V}$_I_, inspired minute ventilation.

### 3.2 Heart rate response to transient hypoxia

**FIGURE 1:**
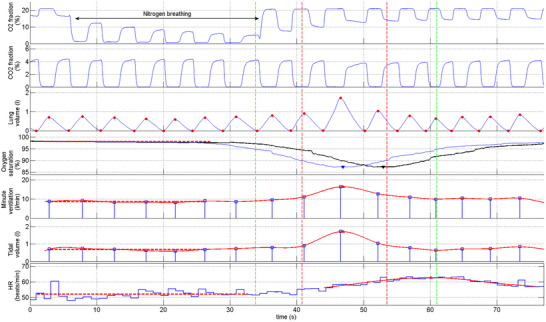
Example of HR response to transient hypoxia. From top to bottom: O_2_ and CO_2_ fractions at the mouth; lung volume with end‐inspiratory and end‐expiratory points (red dots); oxygen saturation at the ear (black continuous line) and estimated oxygen saturation at carotid chemoreceptors (blue continuous line; see main text for details); inspired minute ventilation (blue squares) with 10 Hz cubic spline interpolation (red continuous line); tidal volume (same plot as minute ventilation); beat‐to‐beat HR (blue step waveform) with cubic spline interpolation (red continuous line). Red dashed lines indicate baseline values (mean of raw data). Arrows pointing upwards/downwards indicate  peak/nadir of relevant signals. Abbreviation: HR, heart rate.

Figure 1 shows a representative example of the HR response to transient hypoxia, together with all the relevant signals recorded. The inspired minute ventilation ($\dot{V}$_I_) began to increase relative to the baseline level after the estimated $S_{p}O_{2}$ at the carotid chemoreceptors fell below 95%. After the peak, there was a slow decline towards baseline values. The HR also began to increase after the estimated $S_{p}O_{2}$ fell below 95% but continued to increase after the peak of $\dot{V}$_I_ and the nadir of estimated $S_{p}O_{2}$ were reached. The time course of tidal volume (V_T_) was very similar to that of $\dot{V}$_I_. After the peak, HR decreased slowly, typically at a slower rate than the decrease in $\dot{V}$_I_. In some patients, the respiratory sinus arrhythmia tended to fade in the region of the peak (Figure 1).

In two patients (numbers 2 and 6), the increase/decrease in $\dot{V}$_I_ before and after the peak were not monotonic but fluctuating. A similar pattern of variation was observed for $V_{T}$. Moreover, in one of these patients, there was no increase in $V_{T}$. Despite these peculiar ventilatory patterns, HR began to increase after the estimated $S_{p}O_{2}$ fell below 95% and continued to increase after the peak of $\dot{V}$_I_ and the nadir of estimated $S_{p}O_{2}$ were reached, as seen in the other four patients.

Individual mean changes in $S_{p}O_{2}$, ventilatory parameters and HR induced by the hypoxic stimulus in the two most desaturating transient hypoxia tests are shown in Table 2 (alternative summary statistics are presented in Table A2 in the Appendix). The decrease in $S_{p}O_{2}$ was similar between patients, with a mean value of approximately −11%, whereas the increases in HR, $\dot{V}$_I_ and $V_{T}$ were more variable. The normalized change in HR (ΔHR/Δ$S_{p}O_{2}$) was not related to the hypoxic ventilatory response (ρ = 0.26, *P* = 0.623) nor to the normalized $V_{T}$(Δ $V_{T}$ /Δ $\mathrm{Sp}O_{2}$) (ρ = 0.77, *P* = 0.072).

**TABLE 2:** Individual mean change from baseline (Δ) in oxygen saturation, ventilatory parameters and heart rate in the two most desaturating transient hypoxia tests amongst those with oxygen saturation nadir of <90%.

Patient no.	Δ$S_{p}O_{2}$ ^a^ (%)	Δ$\dot{V}$_I_ ^b^ (L/min)	Δ$\dot{V}$_I_ ^b^ (%)	Δ*V*_T_ ^b^ (L)	Δ*V*_T_ ^b^ (%)	ΔHR^b^ (beats/min)	ΔHR^b^ (%)
1	−10.3	7.5	92.2	1.04	155.4	11.3	21.6
2	−13.2	1.7	10.0	−0.07	−5.7	2.4	4.5
3	−10.5	7.2	89.3	0.65	64.3	2.6	4.2
4	−8.6	4.0	62.2	0.36	69.3	8.5	17.7
5	−9.7	7.3	74.3	0.61	87.6	4.0	7.0
6	−12.5	3.2	28.9	0.16	22.5	2.7	4.7
Mean ± SD	−10.8 ± 1.7	5.3 ± 2.4	59.5 ± 33.4	0.46 ± 0.39	65.6 ± 55.7	5.2 ± 3.7	10.0 ± 7.7

^a^ Difference between hypoxia‐induced nadir and mean baseline.

^b^ Difference between hypoxia‐induced peak and mean baseline.

Abbreviations: HR, heart rate; $S_{p}O_{2}$, oxygen saturation by pulse oximetry with an ear probe; $\dot{V}$_I_, inspired minute ventilation; *V*_T_, tidal volume.

The time delay between the peak of $V_{T}$ and that of HR could not be measured in the patients who had no increase in $V_{T}$ (Table 2, patient 2). In the remaining patients, the delay was 7.9 ± 4.8 s [median (quartile 1, quartile 3): 8.0 (6.3, 11.7) s].

### 3.3 Heart rate response during CSR

One patient (patient 3) developed atrial fibrillation during the night and therefore HR could not be measured. Polysomnographic data of the remaining five patients are shown in Table 3. Individual mean changes in $S_{p}O_{2}$, uncalibrated lung volume and HR during CSR‐CSA are shown in Table A3 in the Appendix. The percentage change in lung volume was very high (297% ± 39%). Despite reduced desaturations (−6.2% ± 2.3%) compared with those observed during the two most desaturating transient hypoxia tests, the HR increases were much higher (12.7 ± 2.9 beats/min). The time delay between the peak of lung volume and the peak of HR was 1.0 ± 0.9 s.

**TABLE 3:** Sleep study results (*n* = 5).

Parameter	Value
TST (min)	312 ± 84
N1 (% TST)	19 ± 7
N2 (% TST)	53 ± 8
N3 (% TST)	11 ± 7
R (% TST)	17 ± 4
Arousal index (events/h)	22.4 ± 6.6
AHI (events/h)	31.3 ± 15.0
AI (events/h)	9.8 ± 7.7
ODI (events/h)	36.8 ± 13.0
T90 (%)	13.4 (4.0, 28.9)
Minimum oxygen saturation (%)	84.4 ± 2.6

*Note*: Data are expressed as the mean ± SD or median (quartile 1, quartile3).

Abbreviations: AHI, apnoea–hypopnoea index; AI, apnoea index; N1, N2, N3, R, sleep stages NREM 1, NREM 2, NREM 3, REM; ODI, 3% oxygen desaturation index; T90, proportion of TST with an oxygen saturation < 90%; TST, total sleep time.

## 4 DISCUSSION

In this study, we used the experimental model of the transient hypoxia test to evaluate the effect of hypoxia on HR in HF patients with CSR‐CSA. We found that HR started to increase around the onset of the ventilation increase, but peaked with a significant delay after the peak in ventilation. The same relationship was found between HR and *V*_T_. Conversely, during the hyperpnoeic phases of CSR‐CSA, the peak in HR was almost synchronous with the peak in lung volume. In addition, the increase in lung volume relative to normal breathing was much greater than that found during transient hypoxia in the laboratory compared with baseline.

### 4.1 Known mechanisms contributing to hypoxia‐induced tachycardia in animals and humans

Several early studies in spontaneously breathing subprimates during hypoxic stimulation of the carotid bodies have consistently shown that when the lungs are denervated and $P_{a}CO_{2}$ is held constant or ventilation artificially kept constant, the chemoreceptor‐induced tachycardia reverts to bradycardia (Daly, 1986; Marshall, 1994). Given that stimulation of pulmonary stretch receptors by increasing lung volume causes reflex tachycardia, it was concluded that the primary effect of carotid body stimulation is bradycardia but that this is overridden by the pulmonary inflation reflex occurring secondarily as a result of the concomitant hyperventilation (Daly, 1986; Marshall, 1994). However, studies in humans have not supported either a primary bradycardic effect of carotid body stimulation (Simon et al., 1995) or that a pulmonary inflation reflex is required for the tachycardic response to hypoxia (Niewinski et al., 2014; Paleczny et al., 2019; Siebenmann et al., 2019; Simon et al., 1995). However, the latter reflex might contribute to tachycardia in some subjects (Simon et al., 1995).

The increase in central inspiratory neural activity during inspiration is known to increase cardiac efferent sympathetic activity and inhibit cardiac efferent vagal activity through a centrally mediated link (Marshall, 1994; Guyenet, 2014). Therefore, it is widely accepted that during sustained stimulation of chemoreceptors by hypoxia, the resulting further increase in central inspiratory activity would contribute to increased HR both in animals and in humans (Daly, 1986; Marshall, 1994; Simon et al., 1995). In addition, animal studies suggest that pulmonary stretch receptors might also have a direct effect on cardiac sympathetic activity by counteracting the effect of increased central inspiratory drive (Marshall, 1994). Therefore, if the pulmonary inflation reflex is weak, the increase in central inspiratory drive would be expected to play a key role in the development of hypoxia‐induced tachycardia.

Stimulation of aortic chemoreceptors in artificially ventilated dogs has been shown to cause tachycardia, which is sympathetically mediated (Karim et al., 1980). Although there is no direct or indirect evidence for this mechanism in humans, it has long been postulated by different investigators to explain hypoxia‐induced tachycardia (Eckberg et al., 1982; Niewinski et al., 2014; Paleczny et al., 2019; Simon et al., 1995).

Studies in rodent models have shown that rostral ventrolateral medulla presympathetic neurons might act as central oxygen sensors or might be activated indirectly by the release of ATP and ‐l‐Lactate from neighbouring astrocytes in response to tissue hypoxia, thereby increasing vasomotor and cardiac sympathetic activity (Reis et al., 1994; Simpson et al., 2024). However, the concept that the CNS $P_{a}O_{2}$ is a physiological regulator of breathing and circulation remains controversial (Guyenet, 2014).

Arterial baroreceptor unloading resulting from hypoxia‐induced vasodilatation has been proposed as a potential mechanism contributing to tachycardia after hypoxic exposure, but experimental evidence has not supported this hypothesis in humans (Paleczny et al., 2019; Simon et al., 1995).

### 4.2 Possible mechanisms underlying the HR response to transient hypoxia in HF patients with CSR‐CSA

Whatever the mechanisms involved in the increase in HR during the transient hypoxia test, the time course of this increase is influenced by the dynamics of vagal and sympathetic control of HR, namely the transfer characteristics between changes in cardiac efferent vagal and sympathetic activity and consequent changes in HR. The HR response to a change in vagal activity is very fast [the response to vagal stimulation in the dog has a latency of ∼0.2 s and can be modelled by a first‐order system with a time constant of ∼1.0–2.5 s (Katona et al., 1970)]. Conversely, the HR response to a change in sympathetic activity is comparatively much slower [latency ∼3 s, time constant ∼4 s (Warner & Cox, 1962)]. We found that, on average, the HR peak occurred ∼8 s after the $V_{T}$ peak. In addition, the increase in $V_{T}$ was modest or absent in two subjects. It is therefore unlikely that the vagally mediated pulmonary inflation reflex was a major determinant of the hypoxia‐induced increase in HR. This conclusion is consistent with the findings of Paleczny et al. (2019) in healthy subjects.

The rising phase of the HR paralleled the falling phase of the estimated oxygen saturation at the chemoreceptors, but it started with a small latency and peaked after the saturation nadir had been reached. This pattern of HR resembles the output of a system consisting of the cascade of a time delay and a first‐order low‐pass filter, where the input to the system is the magnitude of the oxygen desaturation at the chemoreceptors (e.g., 4% when the oxygen saturation level falls by 4% below the baseline level). Therefore, assuming that the increase in central inspiratory drive induced by the hypoxic stimulus reflects the magnitude of the concomitant oxygen desaturation at the chemoreceptors and that this increase causes a corresponding increase in cardiac efferent sympathetic activity, and given that changes in cardiac efferent sympathetic activity are mediated by the slow transfer characteristics of the sinoatrial node, we speculate that the pattern of HR response to transient hypoxia might be predominantly attributable to the sympathetically mediated tachycardic effect of the increased central inspiratory drive. A simple computer simulation described in the Appendix supports this speculation. This mechanism might also have been enhanced by the presence of a weak pulmonary inflation reflex, because this condition would reduce the inhibitory effect of the reflex on sympathetic activation induced by the increase in the central inspiratory drive. Alternatively, it can also be speculated that the HR response to transient hypoxia might be attributable to sympathetic activation induced by brain tissue hypoxia (Simpson et al., 2024). This might explain the previous finding that in HF patients with an abnormally high ventilatory response to hypoxia, the hypoxia‐induced HR increase persisted after carotid body removal (Niewinski et al., 2014).

Although the HR response to aortic chemoreceptor stimulation is sympathetically mediated (Karim et al., 1980) and therefore subject to the delay and distortion effects described above, it seems unlikely that aortic chemoreceptors contributed to the HR response observed in our patients, because these receptors detect the decrease in $P_{a}O_{2}$ induced by hypoxia before the carotid chemoreceptors do, and HF patients have a long circulatory delay (Pinna et al., 2000).

Regarding the HR response during CSR‐CSA, the finding that the increase in HR was almost synchronous with the increase in lung volume suggests that there were different predominant mechanisms mediating this increase in comparison to transient hypoxia in the laboratory. During CSR‐CSA, chemoreceptors are stimulated by the combination of hypoxia and hypercapnia, resulting in a greater increase in ventilation in comparison to hypoxia alone, and therefore a greater increase in central inspiratory neural drive and pulmonary stretch receptor activity (Marshall, 1994). Lung stretch would be particularly pronounced during CSR‐CSA owing to the concomitant increase in ventilation and end‐expiratory lung volume during cyclic hyperventilation phases, leading to large increases in lung volume (Brack et al., 2005). Indeed, we found a far higher percentage increase in lung volume during CSR‐CSA than during transient hypoxia. This would result not only in much greater inhibition of cardiac vagal activity produced by the pulmonary inflation reflex but also in a greater direct effect of lung stretch receptors on cardiac sympathetic activity, opposing its activation induced by the increase in the central inspiratory drive. Therefore, we speculate that the fast vagally mediated pulmonary inflation reflex in combination with the direct effect of pulmonary stretch receptors on cardiac sympathetic activity might be the predominant mechanism mediating the cyclic increase in HR during CSR‐CSA.

### 4.3 Study limitations

The enrolment period of this study was before the advent of angiotensin receptor/neprilysin inhibitor (ARNI) therapy, which has been shown to activate the parasympathetic nervous system significantly in patients with HF and reduced ejection fraction (Boehmer et al., 2024). Because our patients did not receive ARNIs, we cannot exclude the possibility that if ARNIs had been given, the HR response to transient hypoxia would have been affected by this treatment.

## 5 CONCLUSION

This study suggests that the pulmonary inflation reflex is not a key mechanism mediating the tachycardia induced by transient hypoxia alone in HF patients with CSR‐CSA, but is likely to be a key mechanism mediating the cyclic increases in HR during CSR‐CSA. The time course of the HR response to transient hypoxia suggests that a predominant role in this response might be played by the increase in cardiac sympathetic activity resulting from an increased central inspiratory drive or from brain tissue hypoxia. The same time course also makes it unlikely that aortic chemoreceptors are important mediators of HR changes.

## AUTHOR CONTRIBUTIONS

Conceptualization: Gian Domenico Pinna. Investigation: Gian Domenico Pinna, Elena Robbi, Maria Teresa La Rovere and Roberto Maestri. Data curation: Gian Domenico Pinna, Elena Robbi, Maria Teresa La Rovere and Roberto Maestri. Formal analysis: Gian Domenico Pinna. Writing–original draft: Gian Domenico Pinna. Writing–review & editing: Gian Domenico Pinna, Elena Robbi, Maria Teresa La Rovere and Roberto Maestri. Software development: Roberto Maestri. All authors have read and approved the final version of this manuscript and agree to be accountable for all aspects of the work in ensuring that questions related to the accuracy or integrity of any part of the work are appropriately investigated and resolved. All persons designated as authors qualify for authorship, and all those who qualify for authorship are listed.

## CONFLICT OF INTEREST

None declared.

6

**FIGURE A1:**
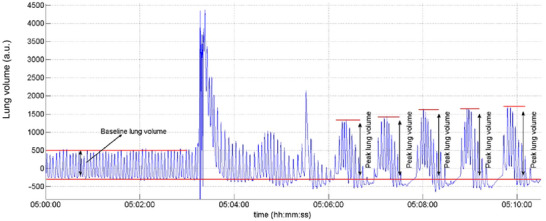
Example of estimation of baseline lung volume and peak lung volume during overnight CSR‐CSA. Details are given in the text. Lung volume was obtained by summing the thorax and abdomen movement signals obtained by respiratory inductance plethysmography and was therefore uncalibrated. Abbreviation: a.u., arbitrary units.

**FIGURE A2:**
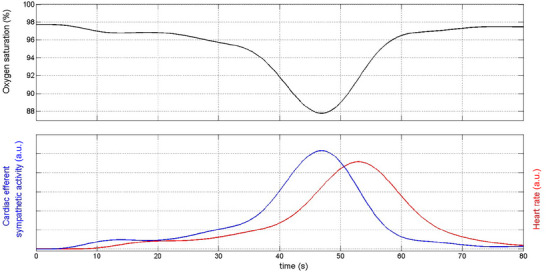
Top panel, oxygen saturation during the transient hypoxia test in a study patient. Bottom panel, blue line indicates the pattern of hypothesized cardiac efferent sympathetic activity (i.e., the input of the system) having the same shape of the magnitude of oxygen desaturation. Bottom panel, red line indicates the pattern of HR (i.e., the output of the system). The delay between the peak of sympathetic activity and that of HR is 6.4 s. Abbreviations: a.u., arbitrary units.

**TABLE A1:** I Alternative summary statistics of Table 1 of the main text.

Patient no.	HVR (L/min/%$S_{p}O_{2}$)	$S_{p}O_{2}$ nadir (%)	$\dot{V}$_I_ peak (L/min)
1	−0.62	87.2	16.5
2	−0.33	82.9	20.4
3	−0.72	85.9	15.9
4	−0.47	88.0	13.5
5	−1.03	84.9	18.4
6	−0.22	83.7	14.6
Median	−0.55	85.4	16.2
(Q1, Q3)	(−0.72, −0.33)	(83.7, 87.2)	(14.6, 18.4)

Abbreviations: HVR, hypoxic ventilatory response; Q, quartile; $S_{p}O_{2}$, oxygen saturation by pulse oximetry with an ear probe $;\;\dot{V}$_I_, inspired minute ventilation.

**TABLE A2:** I Alternative summary statistics of Table 2 of the main text.

Patient no.	Δ$S_{p}O_{2}$ ^a^ (%)	Δ$\dot{V}$_I_ ^b^ (l/min)	Δ$\dot{V}$_I_ ^b^ (%)	Δ*V*_T_ ^b^ (l)	Δ*V*_T_ ^b^ (%)	ΔHR^b^ (beats/min)	ΔHR^b^ (%)
1	−10.3	7.5	92.2	1.04	155.4	11.3	21.6
2	−13.2	1.7	10.0	−0.07	−5.7	2.4	4.5
3	−10.5	7.2	89.3	0.65	64.3	2.6	4.2
4	−8.6	4.0	62.2	0.36	69.3	8.5	17.7
5	−9.7	7.3	74.3	0.61	87.6	4.0	7.0
6	−12.5	3.2	28.9	0.16	22.5	2.7	4.7
Median	−10.4	6.0	68.1	0.48	66.8	3.3	5.8
(Q1, Q3)	(−12.4, −9.7)	(3.2, 7.3)	(29.0, 89.3)	(0.16, 0.65)	(22.5, 87.6)	(2.7, 8.5)	(4.5, 17.7)

^a^ Difference between post‐hypoxic nadir and mean baseline.

^b^ Difference between post‐hypoxic peak and mean baseline.

Abbreviations: HR, heart rate; Q, quartile; $S_{p}O_{2}$, oxygen saturation by pulse oximetry with an ear probe; $\dot{V}$_I_, inspired minute ventilation; *V*_T_, tidal volume.

**TABLE A3:** Individual mean changes (Δ) in oxygen saturation, uncalibrated lung volume and heart rate during CSR‐CSA.

Patient	Peak $S_{p}O_{2}$ ^a^ (%)	Nadir $S_{p}O_{2}$ ^b^ (%)	Δ$S_{p}O_{2}$ ^c^ (%)	ΔLV^d^ (%)	Peak HR^e^ (beats/min)	Nadir HR^f^ (beats/min)	ΔHR^g^ (beats/min)
1	94.5	89.9	−4.6	247	52.8	40.6	12.2
2	96.7	91.9	−4.8	354	52.0	42.3	9.7
3							
4	96.2	86.0	−10.2	310	60.7	43.5	17.2
5	96.0	90.7	−5.3	283	64.3	50.8	13.5
6	97.2	91.2	−6.0	289	56.9	46.1	10.8
Mean ± SD	96.1 ± 1.0	89.9 ± 2.3	−6.2 ± 2.3	297 ± 39	57.3 ± 5.2	44.7 ± 4.0	12.7 ± 2.9
Median	96.2	86.0	5.3	289	56.9	43.5	12.2
(Q1, Q3)	(96.0, 96.7)	(89.9, 91.2)	(−6.0, −4.8)	(283, 310)	(52.8, 60.7)	(42.3, 46.1)	(10.8, 13.5)

^a^ Mean peak of $S_{p}O_{2}$.

^b^ Mean nadir of $S_{p}O_{2}$.

^c^ Mean difference between nadirs and peaks of $S_{p}O_{2}$ (i.e., average magnitude of cyclic desaturations).

^d^ Mean percentage difference between peaks of uncalibrated lung volume and mean baseline lung volume.

^e^ Mean peak of HR.

^f^ Mean nadir of HR.

^g^ Mean difference between peaks and nadirs of HR (i.e., the average magnitude of cyclic HR increases).

Abbreviations: HR, heart rate; LV, lung volume; Q, quartile; $S_{p}O_{2}$, oxygen saturation by pulse oximetry with an ear probe; $\dot{V}$_I_, inspired minute ventilation.

## Data Availability

The data that support the findings of this study are available on request from the corresponding author (G.D.P.).

## Assessment of the ventilatory response to hypoxia by the transient hypoxia method

Prior to the study, all subjects fasted for >2 h and abstained from coffee or tea for 12 h. Subjects breathed with a nose clip in place, through a mouthpiece connected in series to a pneumotachograph and a two‐way, Y‐shaped non‐rebreathing valve, the inspiratory line of which was connected to an inflatable balloon‐type automated breathing valve that allowed switching from room air to a gas bag containing 100% nitrogen. We recorded O_2_ and CO_2_ fractions at the mouth, respiratory airflow by penumotacograph, arterial oxygen saturation using a fast‐response pulse oximeter with an ear probe ($S_{p}O_{2}$), and ECG. The O_2_ and CO_2_ fractions, lung volume and $S_{p}O_{2}$ were displayed in real time together with breath‐by‐breath values of inspired minute ventilation ($\dot{V}$_I_), tidal volume ($V_{T}$)and respiratory rate. Monitoring of ECG and HR was performed with an electrocardiograph. Once the ventilatory parameters were stabilized, the inspiratory line was surreptitiously switched to the gas bag during the expiratory phase and maintained until the subject had taken two breaths of nitrogen. The test was then repeated, randomly increasing and decreasing the number of nitrogen breaths to cover an $S_{p}O_{2}$ range down to ∼80% as evenly as possible, for a total of eight tests, if tolerated by the patient. Each repetition was preceded by a period of air breathing during which respiratory parameters and HR were allowed to return to baseline values.

Signal analysis was performed off‐line. For each test, the $S_{p}O_{2}$ signal was shifted back 1.5 s to compensate for the intrinsic delay of the measuring device, and its nadir was identified. The corresponding peak of $\dot{V}$_I_ was identified within a time window of ±8 s around the $S_{p}O_{2}$ nadir. The hypoxic ventilatory response was calculated as the slope of the least‐squares regression line between all collected pairs of peak $\dot{V}$_I_ and $S_{p}O_{2}$ nadir, including the corresponding baseline values (mean values of prehypoxic $\dot{V}$_I_ and $S_{p}O_{2}$) (Paleczny et al., 2019; Tubek et al., 2016).

## Estimation of the oxygen saturation at carotid chemoreceptors from the $S_{p}O_{2}$ signal recorded at the ear

This estimation was based on the following assumptions: (1) the ventilatory response to hypoxia is predominantly mediated by carotid chemoreceptors (Teppema&Dahan, 2010); (2) the latter respond very rapidly to the change in $P_{a}O_{2}$ of the blood perfusing them (Fidone & Gonzalez, 1986) and sense this change before the same arterial blood reaches the earlobe; (3) the $S_{p}O_{2}$ signal is a reasonable approximation of intra‐arterial oxygen saturation; and (4) the distortion of the oxygen saturation waveform between the carotid bodies and the ear lobe attributable to mixing effects in the vasculature is negligible (Pinna et al., 2000). It follows from these assumptions that the nadir of the estimated $S_{p}O_{2}$ signal would be almost synchronous with the peak of the $\dot{V}$_I_ signal. Accordingly, the $S_{p}O_{2}$ signal at the carotid chemoreceptors was estimated by shifting the $S_{p}O_{2}$ signal at the ear backwards until its nadir was temporally coincident with the peak of the $\dot{V}$_I_ signal.

## Estimation of relevant ventilatory, oxygen saturation and HR parameters during CSR‐CSA

For each patient, four to six segments containing at least three consecutive cycles of CSR‐CSA were selected in the polysomnogram. An example is shown in Figure A1. For each segment, the closest preceding segment (>1 min) of normal breathing was also selected. For each pair of  segments (CSR‐CSA + normal breathing), breath‐by‐breath end‐inspiratory lung volume (EILV) points were identified and measured at the peak of each CSR‐CSA cycle and during normal breathing. Breath‐by‐breath end‐expiratory lung volume (EELV) points during normal breathing were also identified and measured. The difference between mean breath‐by‐breath EILV and mean breath‐by‐breath EELV during normal breathing was used as an estimate of baseline lung volume, whereas the difference between mean peak EILV during CSR‐CSA and mean breath‐by‐breath EELV during normal breathing was used as an estimate of the peak lung volume developed during the hyperpnoeic phases of CSR‐CSA. The latter estimate is based on the fact that during hyperpnoeic phases of CSR‐CSA, there is often a concomitant increase in EELV (Brack et al., 2005) and therefore the difference between EILV and EELV points underestimates the lung volume. Accordingly, the difference between the mean peak lung volume and the mean baseline lung volume was used as an estimate of the lung volume change during CSR‐CSA.

We also calculated the mean difference between nadirs and peaks of $S_{p}O_{2}$ during CSR‐CSA (i.e., the average magnitude of cyclic desaturations) and the mean difference between corresponding peaks and nadirs of HR (i.e., the average magnitude of cyclic HR increases). Finally, within the same segments, the mean delay between the lung volume peaks and the corresponding HR peaks was calculated. This procedure was repeated for all segments, and the results were averaged to obtain final estimates for each patient. Results are shown in Table A3.

## Computer simulation of the hypothesized relationship between cardiac efferent sympathetic activity and HR during the transient hypoxia test

The transfer characteristics between changes in cardiac efferent sympathetic activity and consequent changes in HR were modelled by a system consisting of a cascade of a time delay (3 s) and a first‐order low‐pass filter (time constant: 4 s) (Ten Voorde, 1992; Van de Vooren et al., 2007). Assuming that the increase in central inspiratory drive induced by the hypoxic stimulus and the consequent increase in cardiac efferent sympathetic activity reflect the magnitude of the oxygen desaturation at the carotid chemoreceptors, the output of the system is a delayed and distorted version of the incoming sympathetic outflow. An example is shown in Figure A2.
